# Foamable pluroleosomes system loaded with amlodipine as a repurposed antibacterial topical formulation against MRSA-induced infection; optimization, *in-vitro*, *ex-vivo*, and *in-vivo* studies

**DOI:** 10.1016/j.ijpx.2025.100406

**Published:** 2025-09-24

**Authors:** Alaa S. Eita, Amna M.A. Makky, Asem Anter, Islam A. Khalil

**Affiliations:** a Department of Pharmaceutics, College of Pharmaceutical Sciences and Drug Manufacturing, Misr University for Science and Technology (MUST), P.O. Box 77, Giza, Egypt; b Department of Pharmaceutics and Industrial Pharmacy, Faculty of Pharmacy, Cairo University, Cairo, Egypt; c Microbiology Unit, Drug Factory, College of Pharmaceutical Sciences and Drug Manufacturing, Misr University for Science and Technology (MUST), P.O. Box 77, Giza, Egypt

**Keywords:** Amlodipine, Pluroleosomes, Foam, Repurposing, MRSA infection, Topical, Open wound

## Abstract

Amlodipine besylate (AML) is a renowned antihypertensive drug currently acknowledged for having antibacterial activity. AML repositioning can be helpful in the defeat of microbial-resistant strains. Loading amlodipine in the pluroleosomes (PLOs) foam system is desired to approach innovative remedies with a convenient application capable of targeting deep infections. The mixture design was employed to generate different pluroleosomes formulations consisting of various ratios of Pluronic F-127, oleic acid, and soya lecithin loaded with amlodipine. Based on the desirability function, the selected optimized formula (AML-PLOs), consisting of 4.875 for lecithin, one for oleic acid, and 1.125 for pluronic, exhibits a particle size of 320.56 ± 15.5 nm, a polydispersity index of 0.4461 ± 0.03, a surface charge of 15.261 ± 0.62 mV, and AML entrapment of 71.25 ± 3.52 %. The morphological image displayed a uniform spherical shape at the nanoscale. In addition, thermal analysis and infrared spectroscopy (IR) proved the suitability of AML-pluroleosome vesicles. Tween 20, the selected nonionic surfactant in foam preparation, achieved the demand values of foam parameters and showed adequate stability upon storage for up to 90 days. The selected AML-PLO foam showed complete AML release after 48 h in a controlled manner, and the cumulative amount permeated after 24 h was about 45 %. Efficient penetration through dermal strata was affirmed by utilizing a confocal microscope. *In vitro* microbiological assay, besides the *in vivo* microbiological and histopathological studies employing a wound healing model, validated the antibacterial efficacy of amlodipine. Those outcomes demonstrated that the prepared pluroleosome foam system of AML is a competent candidate for combating topical bacterial infection.

## Graphical abstract

**Figure:**
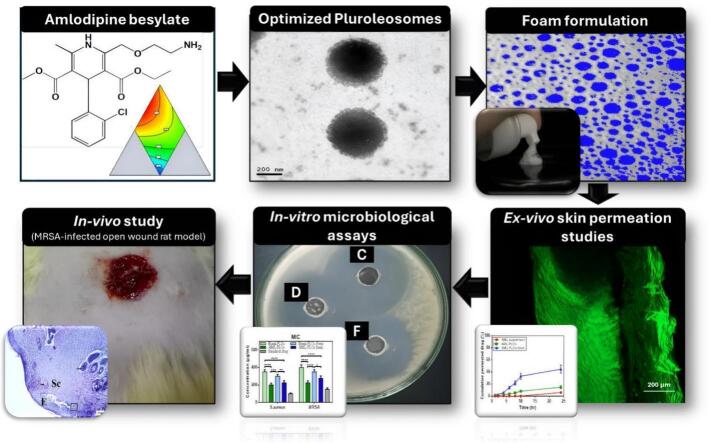

## 1 Introduction

Bacterial skin and soft tissue infections (SSTIs), mainly cellulitis and pyoderma, are considered crucial health issues facing healthcare practitioners globally (Chambers, 2021; Stevens et al., 2014). Open wounds are considered a significant predisposing factor for skin and SSTIs infection, which can result in potential complications (Seaton, 2009). Numerous bacterial species can induce SSTI, chiefly Gram-positive bacteria, including *Staphylococcus aureus*, one of the most prevalent isolated strains. Furthermore, community-acquired methicillin-resistant *Staphylococcus aureus* (MRSA) accounts for almost 59 % of SSTIs declared in emergency departments (Chambers, 2021). Efficient treatment of SSTIs is fundamental to overcoming the illness's potential progression from mild-moderate conditions such as cellulitis and impetigo to a life-threatening status like necrotizing fasciitis (Marques and Abbade, 2020). The effectiveness of antibacterial therapy for skin ailments confronts several challenges owing to the skin barrier, topical formulation features, and antibiotic resistance (Marzouk et al., 2024). The advent of antimicrobial resistance (AMR) to conventional antibiotics demands identifying potential novel therapeutic methods or new chemical entities to overcome resistant issues (Annunziato, 2019). Consequently, ongoing research is pivotal to discovering alternative approaches to resolving those therapeutic challenges.

Discovering a new entity of antibacterial drug is a protracted and costly process that encounters considerable challenges in delivering innovative therapies expeditiously. Drug repurposing provides a suitable strategy to reduce the expense and duration of the drug development procedure by using an approved drug outside the scope of its primary medical use (Boyd et al., 2021). Recently, antibacterial therapy relies on drug repurposing to identify medications with antibacterial activity as alternatives to confront antibiotic resistance (Farha and Brown, 2019). Where *S. aureus* is consistently at the forefront of acquiring antimicrobial resistance, for instance, methicillin resistance (MRSA), diverse drugs are reported by Aggarwal et al. against resistant strains. Drugs from different classes, such as NSAIDs, antidepressants, antirheumatic, antidiabetics, and antiparasitic, are recognized with antimicrobial activity (Aggarwal et al., 2024). Moreover, amlodipine, as an antihypertensive drug, demonstrates an antibacterial property (Hua et al., 2022).

Amlodipine (AML) is well-known as a dihydropyridine calcium channel blocker used to treat cardiovascular disorders by inhibiting calcium influx, resulting in the dilating of smooth muscle of blood vessels (Sheraz et al., 2016). Otherwise, the study by Tatar et al. pronounced the antibacterial activity of AML against different bacterial species (Tatar et al., 2017). Furthermore, Sharma et al., while investigating the impact of AML as an antimicrobial on Pseudomonas, demonstrated AML's potential antibacterial efficacy in diminishing different virulence traits, including biofilm formation and resistance to oxidative stress. The activity of amlodipine as an antibacterial agent is interpreted by disrupting bacterial cell membranes, resulting in the release of biological components, including proteins and nucleic acids. This disruption attenuates the integrity of the bacterial cell, eventually resulting in cell death. (Sharma et al., 2024). In addition, AML inhibits beta-lactamase, which is responsible for antibiotic resistance (Glajzner et al., 2024). Utilizing AML in the repositioning scope as an antibacterial treatment can defeat the resistance issues of traditional antibiotics.

AML, in the dermal application for bacterial skin and wound infection, prefers employing nanocarrier systems to promote permeation, control release, and ensure optimum therapeutic efficacy (Teo et al., 2025). Various nanosystems were utilized in previous investigations as carriers to uphold the healing scope of the infected wound. Metal nanoparticles, comprising gold, silver, and copper, were considered a perfect choice because of their synergistic antibacterial effects, with concern for safety issues, as they can lead to human cell toxicity (Altun et al., 2021; Zhang et al., 2022). Also, previous assessment conducted by Hasan et al. proved the suitability of polymer nanoparticles, particularly PLGA, in improving the efficiency of antibacterial drugs for targeting wound-resistant infection (Hasan et al., 2019). However, the stability of polymers requires further considerations and improvement (Bentaleb et al., 2025). Additionally, lipid-based nanoparticles were recognized as a controlled release system that can enhance antibacterial effect and wound healing. Lipid carriers appear to have synergistic antibacterial impacts and combat resistant bacterial infection (Ryan et al., 2023; Zhou et al., 2023).

Vesicles as versatile lipid- based nanocarriers can enhance transdermal delivery by optimizing solubility of the drug in the formulation, facilitating drug penetration into dermal layers, and reinforcing the depot action of the drug (Chacko et al., 2020). Pluroleosome system fabrication using Pluronic F-127, oleic acid (OA), and soya lecithin establishes a functional vesicle system that acquires the desired features. Lecithin vesicles established a suitable carrier for lipophilic drugs with appropriate encapsulation efficiency and controlled drug release (Khan et al., 2022). The coexistence of a penetration enhancer (OA) and surfactant (pluronic) is in demand to improve vesicle permeation across dermal layers by enhancing skin fluidity and diffusivity (Nayak et al., 2024; Zakir et al., 2010). Previous investigation by Ahmad et al. proved that oleic acid-based vesicles enhance lipid system deformability threefold compared to conventional liposomes, leading to elevated dermal drug penetration (Ahmad et al., 2024). In addition, another investigation mentioned that the hybrid vesicle containing lecithin and pluronic reflects the existence of pluronic preference to enhance stability and drug loading (Parhi et al., 2016). Also, a previous study by Thakur et al. demonstrated the significant potential of a hybrid vesicle loaded with fusidic acid in the struggle against MRSA-infected wounds (Thakur et al., 2019). Furthermore, implementing nanovesicle preparations consistently favored incorporation in convenient topical formulations, particularly cream, gel, or foam systems.

A foam delivery system is a pharmaceutical dosage form of gas bubbles incorporated into a liquid phase containing stabilizing and foaming agents, predominantly surfactants (Hoc and Haznar-Garbacz, 2021). This system is targeted to improve topical drug application and penetration. Restful spreading with low friction, chiefly in contact with sensitive or irritated skin, nominates the foam system over other conventional formulations to seek patient compliance and satisfaction (Myrzagulova et al., 2025). In comparison to hydrogel, as discussed previously by Oliveira and Almeida's study, foam is more preferred in terms of stability, as hydrogel is highly hydrated and the aqueous medium is vulnerable to chemical degradation (Oliveira and Almeida, 2023). Also, foam is more suitable in infected wounds with high exudate to control surplus moisture and maintain a suitable environment for avoiding augmentation in bacterial load and accelerating healing (Nuutila and Eriksson, 2021). Farkas et al. provide two techniques for preparing the foam system: supersaturating gas under pressure to produce aerosol foam or employing a mechanical method of distributing the gas phase into the liquid through agitation. Foam liquid solution created by the mechanical method requires the utilization of a non-propellant pump actuator for foam fabrication (Farkas et al., 2019).

This study planned to elucidate the antibacterial activity of amlodipine (AML) by formulating lipidic vesicles (Pluroleosomes) enriched with soya lecithin, oleic acid, and Pluronic F-127 to enhance AML solubilization and penetration. After that, consolidating pluroleosome (PLOs) into a foam system promotes topical application and dermal deposition. A D-optimal mixture design was applied to systematically assess and optimize AML-PLO formulae to ascertain the most desirable ratio for vesicle composition incorporated in the selected foam system. Various characteristics of the PLOs and foam system were investigated to guarantee the overall suitability and stability of the system. Moreover, *in-vitro* microbiological and *in-vivo* studies were performed to affirm AML as an antibacterial agent.

## 2 Materials and methods

### 2.1 Materials

Amlodipine besylate was a gift from the EVA Pharma company in Egypt. Soya A lecithin was gained from Phospholipid GmbH (Nattermannallee, Germany). Brij 56, Brij 96, Hypromellose (HPMC), Tween 20, Tween 80, Sodium dodecyl sulfate (SDS), Pluronic (F-127), and Cellulose membrane of 12,000 Da as MWCO were obtained from Sigma-Aldrich (St. Loius, MO, USA). Oleic acid, Propylene Glycol, Glycerin, Monopotassium phosphate, and Disodium phosphate were bought from El-Nasr Pharmaceutical Co. (Cairo, Egypt). Additional handled materials were ensured to be of high quality.

### 2.2 Pluroleosome formulation

#### 2.2.1 Amlodipine-loaded pluroleosome

Amlodipine Pluroleosomes (AML-PLOs) were prepared using a thin film hydration technique per the reported method (Verma et al., 2014) with minor changes. According to the employed design after conducting screening study (**Table S1 in supplementary data)**, several AML-PLOs formulae were prepared by utilizing variable amounts of Soya A lecithin (SL), Oleic acid (OA), and Pluronic F-127 upon solubilized the different ratios using a blend of chloroform and methanol with a propotion of 2:1 (*v*/v), and then add a solution of 100 mg amlodipine which was dissolved before in a 10 methanol. The prepared blend was exposed to reduced pressure using a rotary evaporator until complete solvent evaporation was achieved. Afterward, the formed film was hydrated with deionized water (20 mL) and exposed to bath sonication for one minute, followed by gentle mixing for 60 min at room temperature.

#### 2.2.2 Experimental design of pluroleosome

Mixture designs consider independent variables to be the proportions of different components in a mixture. The evaluated response is supposed to depend only on the relative proportions of the ingredients, not on the mixture amount. Therefore, this design is more efficient than the OFAT and RSM approaches (Beg, 2021). This investigation applied a D-optimal mixture design for (AML-PLOs) formulae using Design-Expert® software (Stat-Ease Inc., Minneapolis, MN, USA) to investigate different ratios of study factors. The changeable inputs were lecithin ratio (X_1_), Oleic acid ratio (X_2_), and Pluronic ratio (X_3_). While particle size (PS) (nm, Y_1_), polydispersity index (PDI) (Y_2_), Zeta potential (ZP) (mV, Y_3_), and entrapment efficiency (EE) (%, Y_4_) were measured responses, as clarified in Table 1. Depending on the combination of different variables' levels, 16 trials were produced by the software (Table 3). Optimization suggestions depend on selecting a variable that leads to the most preferred formulation, which owns the minimum PS and PDI with the greatest ZP and EE values.

**Table 1:** Changeable inputs and measured responses adopted in a mixture design for prepared optimal AML-PLOs.

Changeable inputs	Levels	
	(−1)	(1)
X_1_: Soya lecithin ratio	1	5
X_2_: Oleic acid ratio	1	3
X_3_: Pluronic ratio	1	3
**Evaluated responses**	**Desirability constraints**	
Y_1_: Particle size (nm)	Minimize	
Y_2_: Polydispersity index	Minimize	
Y_3_: Zeta potential (absolute value) (mV)	Maximize	
Y_4_: Entrapment efficiency (%)	Maximize	

#### 2.2.3 Amlodipine-loaded pluroleosome characterization

##### 2.2.3.1 Zetasizer analysis

Zetasizer (Malvern Instruments, Malvern, UK) was employed using the technique of dynamic light scattering (DLS) for evaluating zeta potential (ZP), particle size (PS), and polydispersity index (PDI). Pluroleosome dispersion was diluted 50-fold using deionized water; subsequently, the mean and standard deviation for all AML-PLO formulations were computed in triplicate (Abdelalim et al., 2020).

##### 2.2.3.2 Encapsulation efficiency and drug loading

Encapsulation efficiency (percentage of entrapped drug) in the pluroleosome system was evaluated indirectly. One milliliter of the AML-PLOs formula, corresponding to 5 mg AML, was separated at 20000 rpm at 4 °C employing a cooling centrifuge (Sigma 3 K 30, Germany) for one hour. Subsequently, the AML in the supernatant was gathered. Then, the pellet residue was washed and centrifuged twice more to regather the supernatant, as previously mentioned (Eita et al., 2022a), with a slight modification. The consolidated supernatant was measured at 364.5 nm utilizing a UV–Vis spectrophotometer (Shimadzu UV 1650 Spectrophotometer, Japan). EE% was evaluated thrice employing the mentioned equation:

**(1)** $$\mathrm{EE}\%=\frac{T_{\mathrm{AML}}-U_{\mathrm{AML}}}{T_{\mathrm{AML}}}\times 100$$

T_AML_ and U_AML_ symbolize the overall amlodipine and unentrapped amlodipine in the supernatant.

Drug loading related to lipid was calculated thrice for the optimum AML-PLOs using the following equation (Alskär et al., 2016):

**(2)** $$\mathrm{Drug loading}\;\left(\%\frac{w}{w}\right)=\frac{\mathrm{AML}\;\mathrm{amount in PLOs}}{T_{\mathrm{Lipid}}}\times 100$$

T_Lipid_ symbolizes the total amount of lipids used.

#### 2.2.4 Features of optimum AML-PLOs

##### 2.2.4.1 Vesicle morphology

AML-PLOs' structural features were visualized using a transmission electron microscope (TEM; CM12; Philips, USA). The resulting optimal AML-PLO from the mixture design was examined to image the system structure. An appropriate sample dilution was stained with an aqueous solution of 2 % negative phosphotungstic acid stain and dried on a carbon grid before being assessed using TEM at 80 kV (Alyami et al., 2023).

##### 2.2.4.2 Differential scanning calorimetry

Thermal analysis was performed to create the thermograms of pure AML, Soya A lecithin, Oleic acid, Pluronic F-127, and AML-PLOs vesicles using a differential scanning calorimeter (DSC, Shimadzu TA-60, Japan). Roughly 5 mg of each studied sample was sealed in an aluminum pan and scanned at 25 to 250 degrees Celsius. Thermograms were obtained at a scanning rate of 10C/min using nitrogen gas as a purge (Musallam et al., 2022).

##### 2.2.4.3 Fourier transform infrared spectroscopy

The IR spectra of AML, Soya A lecithin, Oleic acid, Pluronic F-127, and the AML-PLOs optimized formula were assessed using Fourier transform infrared spectroscopy (FTIR) (Shimadzu IR-Affinity-1, Japan) to track any drug-excipient incompatibility. Approximately 3 mg of each sample was compacted into a disk after mixing with KBr, and then the scanning was applied in the range of 400–4000 cm^−1^ with a resolution of 4 cm^−1^ at ambient temperature with a speed of 2 mm/s (Elgendy et al., 2023).

### 2.3 Foamable AML-PLO system formulation

#### 2.3.1 AML-PLOs-Foam system fabrication

The non-propellant Foam (NPF) loaded with AML-PLOs was prepared using the optimized foam composition from previous work and the preparation method as reported (Eita et al., 2022b), with slight alterations. A 1.5 % (*w*/*v*) glycerin was used as a stabilizing agent in addition to 0.5 % (w/v) propylene glycol (PG) to levigate 0.5 % of hydroxypropyl methylcellulose (HPMC). 1.249 % (w/v) sodium dodecyl sulfate as an anionic surfactant (SAA) was added to the blend. Subsequently, different formulae were assessed using the same ratio (4 % w/v) of four types of nonionic surfactant (SAA): Tween 20, Tween 80, Brij 56, and Brij 96. Nonionic SAA was mixed in the prepared blend and combined with the AML-PLOs system to evaluate the impact of nonionic SAA types on foam system attributes. The loaded foam system was blended on a magnetic stirrer (model MSH-20D, GmbH, Germany) at 1000 rpm for 60 min at ambient temperature. After that, a polypropylene propellant-free pump was used to prepare foam from the aqueous solution for foam fabrication during inspection or application.

#### 2.3.2 AML-PLOs-Foam system assessment

##### 2.3.2.1 Foam calculated parameters

Particular investigated parameters can be used as a primary method for foam evaluation to estimate the effect of different foam compositions on foamability and stability features (Farkas et al., 2019). One of the most used methods in parameter measurement is the cylinder method. A glass cylinder is filled with a specific volume of the foamable liquid solution, and foam fabrication is done upon applying manual shaking; after a particular time interval, foam volume and drain volume will be observed in addition to the initial foam volume to calculate the intended parameters (Arzhavitina and Steckel, 2010). In this study, the homogenizer was utilized as an alternative method for foam production as per the previous method (Parsa et al., 2019), which was used to reduce the variability of manual foam formation between studied formulations. A 3 mL sample in a falcon tube was homogenized at 13500 rpm for 2 min; foam volume and drain volume were noted to calculate the expansion percentage of the foam (FE), the volume stability (FVS), and the liquid stability (FLS) according to the equations shown in Table 2.

**Table 2:** Equations used for calculating foam parameters.

Calculated parameters (%)	Equations
Foam expansion (FE)	$\mathrm{FE}\%=\frac{\mathrm{Vfm}-\mathrm{Vft}}{\mathrm{Vft}}\times 100$**(Eq.3)**
Foam volume stability (FVS)	$\mathrm{FVS}\%=\frac{\mathrm{Vfm}/30\min}{\mathrm{Vft}}\times 100$**(Eq.4)**
Foam liquid stability (FLS)	$\mathrm{FLS}\%=\frac{\mathrm{Vl}/30\min}{\mathrm{Vft}}\times 100$**(Eq.5)**

Abbreviations: *Vfm*, resulting foam volume (mL); *Vft*, initial solution volume (mL); *Vfm/*30 min, foam volume after 30 min (mL); *Vl/*30 min, drained volume after 30 min (mL).

##### 2.3.2.2 Foam half-life

The Foam half-life is assigned as the time required by the fabricated foam to reach half of its initial volume; a higher half-life value is preferred, as it indicates a more stable foam system (Wang et al., 2017a). As mentioned, the fabricated foam used a homogenizer instead of manual shaking (Parsa et al., 2019).

##### 2.3.2.3 Viscosity

The formulation's liquid viscosity was measured thrice utilizing a Brookfield DV3T Viscometer (Brookfield Engineering Laboratories, Inc., Middleboro, MA) at 250 rpm and a temperature of 25 °C ± 2 °C. Viscosity is a significant concern in foamability and the pump's ability to produce Foam (Derikvand and Riazi, 2016).

### 2.4 Optimum AML-PLOs foam evaluation

#### 2.4.1 AML-PLOs and foam system comparative analysis

PS, PDI, and ZP were re-measured after AML-PLOs were incorporated into the foam formulation to investigate the foam system's applicability and favorability for dermal application.

#### 2.4.2 Prediction of foam bubble size

A foam image was analyzed using ImageJ (U. S. NIH, Maryland, USA) to determine bubble size and size distribution. A picture of the foam system was taken after the foam was fabricated on a glass surface using the NPF pump. The software aided in image adjustment, and then a histogram was depicted to represent bubble size globule mean (μm) *versus* size distribution (Farkas et al., 2019).

#### 2.4.3 Storage impact on PS, PDI, and ZP

Optimized AML-PLOs nanodispersion and foam formulation were retained in sealed, tawny glass vials at 4 ± 1 °C and RT for 3 months to determine system stability during storage. PS, PDI, ZP, and physical appearance were observed to track changes in the formulation's physicochemical stability. Moreover, the production of foam was appraised to validate system appropriateness. Assessments were conducted thrice, primarily after the system preparation and during the monthly evaluation.

#### 2.4.4 *In-vitro* release study

In addition to the AML suspension, the AML release profile from PLOs and PLOs-foam systems was carried out using a modified vertical diffusion cell adapted for foam formulation and the large volume of the receptor (Falusi et al., 2024; Jayadev and Ismail, 2023). A receiver chamber containing 100 mL of PBS (pH 7.4) and ethanol (9:1) as release media was maintained under an adjusted temperature of 32 ± 0.5 °C with uninterrupted mixing at 100 rpm. As reported by Kapoor et al., ethanol is used in the media of AML released from transdermal nano lipid carriers to maintain sink conditions (Kapoor et al., 2019). A sufficient volume of the investigated samples, equal to 10 mg AML, was dropped into a donor compartment of a 5 cm^2^ surface area. At predefined periods, 2 mL of the samples from the receiver chamber were withdrawn and replaced with fresh medium. Withdrawn samples were measured at each time interval employing a spectrophotometer (UV/visible) at 364.5 nm to calculate the amount of released AML.

Data resulting from the release evaluated profile was kinetically assessed by employing various models such as zero order, first order, second order, Higuchi model, and Hixson–Crowell model, *etc.*, looking for the best fitting one with a higher correlation coefficient (R^2^) upon using DDsolver software. Data interpretation was based on the explanation of Costa and Sousa Lobo and Zhang et al. (Costa and Sousa Lobo, 2001; Zhang et al., 2010).

### 2.5 *Ex-vivo* studies

#### 2.5.1 Skin preparation

The *ex-vivo* permeation study was performed under the ethical committee permission of the Department of Pharmacy, Cairo University (Protocol number PI 3305), using rat skin as an animal model for permeation experiments. Albino rats were sacrificed using cervical dislocation, and the skin tissues were collected. A hair clipper was utilized to eliminate the dorsal hair, and then the shaved area was separated using a surgical blade. After the excision, a scalpel was employed to meticulously excise the subcutaneous adipose tissue from the abdominal region of the skin. Subsequently, the skin was washed with saline solution and subdivided into suitable pieces for permeation studies (Uthaman and Koland, 2020).

#### 2.5.2 *Ex-vivo* permeation and deposition study

The *ex-vivo* skin penetration of AML from PLOs and PLOs-foam systems was conducted as reported by Kapoor et al. using a modified diffusion cell with slight alteration (Kapoor et al., 2019). A fresh piece of rat skin was attached amid the compartments of diffusion cells with an area of 5 cm^2^, keeping the stratum corneum side facing the donor compartment. AML-PLOs, AML-PLOs foam system (equal to 10 mg), and 10 mg of drug suspension were put in the donor compartment. In contrast, the receiving compartment was loaded with 100 mL of PBS (pH 7.4) and ethanol (9:1), kept at 37 ± 0.5 °C under continuous mixing at 100 rpm for 24 h. Two mL Aliquots were withdrawn from the receiving compartment at planned intervals and replaced immediately with fresh media. Afterward, the collected samples were filtered (filter membrane with a 0.45 μm) and assessed using a validated HPLC method. The permeated amount (cumulative) from the studied formulae was graphed *versus* time to evaluate the penetration rate. The permeation flux (Jss) parameter was also investigated.

##### 2.5.2.1 Skin deposition

After the completion of the penetration study, the used skin tissues were removed and washed with normal saline, homogenized with a definite volume of methanol, and kept in a 37 °C water bath at 100 rpm for 24 h to ensure adequate extraction of the drug deposited in dermal strata. The supernatant was separated, filtered, and assessed using a validated HPLC method (Abdellatif et al., 2017).

##### 2.5.2.2 HPLC assay

A previously conveyed and validated HPLC method determined by Sankar et al. was used with little modification for AML quantitation in *ex-vivo* studies (Sankar et al., 2006). The HPLC (Agilent Technologies, Santa Clara, CA, USA) with an autosampler was utilized, and the UV detector was set at λ _max_ 364.6 nm. The mobile phase was a mixture of Buffer solution (pH 3) and acetonitrile (50:50, *v*/v) with a one mL per minute flow rate at 40 °C. All samples were filtered with a 0.45 μm millipore filter before injection to prevent column blockage, and a volume of 50 μL was injected into the analytical column from each sample. The analytical method was validated in terms of linearity, specificity, precision, limit of detection, and recovery.

#### 2.5.3 *Ex-vivo* visualization study

In this study, the AML-PLOs system was loaded into the foam formulation, seeking deep penetration and convenient application. Therefore, in the *ex-vivo* visualization study, a fluorescent dye was loaded in the system instead of AML to observe and track the capability of the foam system penetration using confocal laser scanning microscopy (CLSM).

##### 2.5.3.1 FDA-PLO foam system preparation and experiment setup

The thin film method was utilized for dye-loaded pluroleosome preparation per the reported method in section 2.2.1 upon clarified AML-PLOs preparation, considering the optimized selected mixture ratios. However, instead of AML, Fluorescein Diacetate (FDA) dye was loaded in PLO, and subsequently, FDA-PLO was incorporated into the chosen foam system composition. The permeation study was performed for FDA-PLO for 24 h, similar to the whole condition of the conducted *ex-vivo* permeation study. Thereafter, the dermal tissue was collected and washed with normal saline to ensure the removal of any excess dye, and then it was kept at −20 °C until CLSM visualization.

##### 2.5.3.2 Confocal imaging study

A small part of the dermal tissue was investigated using a microscope (LSM 710; Carl Zeiss, Oberkochen, Germany), with an FDA excitation wavelength at 490 nm and an emission wavelength at 529 nm. Considering skin thickness, the observation yielded qualitative and quantitative data regarding the foam system's permeation. Optical scanning was conducted across a depth range of 0 μm to 285 μm, utilizing increments of 15 μm. The penetration attribute of the system within the dermal strata was elucidated by constructing a three-dimensional graphical representation employing a *Z*-stack model.

### 2.6 *In-vitro* microbiological assays

#### 2.6.1 Microbial test cultures

*Staphylococcus aureus* and methicillin-resistant *S. aureus* (MRSA), the standard strains used per standard BSL-2 laboratory safety protocols, were acquired from the culture collection unit at the Regional Center for Mycology and Biotechnology in Al Azhar University. Bacterial strains were preserved in 15 % (*v*/v) glycerol stocks at −80 °C. A single colony of tested strains was grown in Brain Heart Infusion (BHI) broth. Afterward, the bacteria were centrifuged for 15 min at 10000 ×*g*, washed, and resuspended in sterile phosphate-buffered saline (PBS). The bacterial suspension was set at a final count of 2.6 × 10^5^ colony-forming units CFU/mL. Isolated strains were preserved at 37 °C on mannitol salt agar (MSA). Strain purity was consequently ensured by observing cell morphology (Gram stain), colony morphology, and biochemical tests (catalase and coagulase), relying on standard procedures.

#### 2.6.2 Agar well diffusion method

The Kirby-Bauer technique was employed for antibacterial screening, relying on the Clinical Laboratory Standard Institutions (CLSI) guidelines for agar well diffusion (CLSI, 2020). A volume of 100 μL from the selected bacterial suspension, adjusted to a concentration of 1.5 × 10^8^ CFU/mL, was evenly spread onto sterile petri dishes containing 25 mL of Mueller-Hinton Agar (MHA). The inoculated plates were incubated overnight at 37 °C. Following incubation, bacterial inocula were prepared using the direct colony suspension method, per antimicrobial susceptibility testing protocols. Well-isolated colonies were aseptically collected using a sterile wire loop and suspended in sterile 0.85 % saline solution. The turbidity of the resulting suspension was adjusted to equal the 0.5 McFarland standard, ensuring consistency in bacterial concentration for downstream assays. Subsequently, the bacterial cultures were evenly spread across the surface of MHA using sterile swabs. Wells with a diameter of 5 mm were created in the inoculated agar using a sterile cork borer. Each well was then filled with 50 μL of the appropriate dilution of the tested formulation, prepared at a concentration of 3.3 mg/mL. The commercially available Mupirax® ointment served as a standard control. The agar plates were cultured at 37 °C for 24 h, and the inhibition attributes were determined by measuring the apparent zone diameter around the well using a ruler scale. The study was executed in triplicate to verify the findings statistically.

#### 2.6.3 Micro-dilution assay (MIC/MBC estimation)

The minimum inhibitory concentration (MIC) was determined using the broth microdilution method under the recommendation of the CLSI guideline (CLSI, 2023). Two-fold serial dilutions of the AML-free drug and each tested formulation were prepared in sterile, capped universal bottles under aseptic conditions. Subsequently, 2 mL of overnight bacterial cultures of *S. aureus* and MRSA (incubated at 37 °C) were mixed with 2 mL of each dilution. The mixtures were vortexed briefly to ensure homogenization and then incubated at 37 °C for 18 h to assess bacterial growth inhibition. An additional 2 mL of broth was prepared in a separate universal bottle, inoculated with *S. aureus* and MRSA, and stored overnight at 4 °C. This preparation served as the growth control for assessing complete inhibition. The positive control consisted of Mueller-Hinton Broth (MHB) inoculated only with the bacterial suspension, while Mupirax® was used as a standard control. Subsequently, relying on growth turbidity, the MIC values were stated as the lowest formulation concentration inhibiting the growth of selected strains. Relative to the positive control, the MIC was assessed to evaluate the inhibitory action of the tested formulations against the bacterial strains (Balouiri et al., 2016; Li and Ma, 2024).

Immediately after MIC determination, aliquots from tubes exhibiting no visible growth were used to assess the minimum bactericidal concentration (MBC). Approximately 100 μL of each inhibitory culture was spread onto sterile nutrient agar plates and incubated at 37 °C for 24 h. The MBC was the lowest concentration at which no bacterial colonies grew on the agar (Ranjutha et al., 2023).

### 2.7 *In-vivo* study

#### 2.7.1 Microbiological assay

The *in-vivo* study assessed the antibacterial activity of AML and the influence of AML-loaded pluroleosome foam formulation against the Methicillin-resistant *S. aureus* (MRSA) strain using an MRSA infection on an open wound rat model. This investigation was executed in a manner consistent with the guidelines authorized by the College of Pharmacy's ethical committee, Cairo University (serial number of the protocol PI 3305). Sixteen male rats (Wistar albino), about 200–250 g in weight, enrolled in the study were assigned to four groups (*n* = 4) and housed individually for animal preparation with unrestricted access to food and water. The first group was assigned to the infected wound with no treatment (positive control). The second group was treated with a once-daily dose of the standard antibacterial drug (Muiprax®). The third group was assigned to the AML-PLOs foam formulation with a single dose every 48 h. The fourth group was set to the blank pluroleosome foam formula. The rat dorsal skin was shaved and then injected subcutaneously with betamethasone (2 mg/kg body weight/day) thrice daily to suppress rat immunity. A circular skin incision with a diameter of 20 mm of the dorsal skin was excised using sterile scissors, and the incision was left for 24 h to allow for wound development. Afterward, wound infection was induced by 100 μL of the MRSA strain suspended in sterile saline (2.6 × 10^5^ CFU/mL). Signs of bacterial wound infection were observed 24 h after bacterial induction, and thereafter, treatment was initiated. The wounds received 100 μL of each tested group's formulation *via* topical administration for 21 days. The treated wounds and controls were measured at 5, 7, 14, and 21 days post-treatment for each group. At each time point, rats were euthanized by cervical dislocation. Skin specimens were gathered from the tested groups for microbiological and histopathological assessment. For the microbial evaluation, homogenized skin samples were cultured in mannitol salt agar (MSA) for 24 h at 37 °C, followed by colony-forming unit (CFU) enumeration (El-Shiekh et al., 2021; Salem et al., 2021).

#### 2.7.2 Wound closure

At each time point, wound images were captured with a metric ruler placed adjacent to the wound to provide a scale reference and enable accurate estimation of the wound area through ImageJ analysis software (U. S. NIH, Maryland, USA). The wound closure percentage was mathematically calculated using the mentioned equation (Ehterami et al., 2018).

**(6)** $$\mathrm{Wound closure}\;\left(\%\right)=\frac{\mathrm{Initial wound area}-\mathrm{open wound area}}{\mathrm{Initial wound area}}\times 100$$

#### 2.7.3 Histopathological dissection

The histopathological inspection was performed on the rat dorsum skin specimens at each time interval for all groups. Skin specimens were washed, fixed for 24 h in 10 % buffered formalin, rinsed, dehydrated, trimmed, and embedded in paraffin. Sequential 5–7 μm-thick skin sections were cut and then affixed to glass slides. The acquired tissue sections were deparaffinized using xylol and stained using hematoxylin and eosin (H&E) for histopathological evaluation through the electron light microscope (Bancroft and Layton, 2019).

The scoring system was employed to assess the various wound healing stages at different time intervals, depending on the age of the wound. Wound tissue sections were evaluated based on the subsequent criteria: Scab, inflammatory cell infiltration, re-epithelialization, granulation, and scar tissue formation. The parameters mentioned above were classified as follows: (−) = normal histology (no changes), (+) ≤25 % (mild alterations), (++) = 25 %–50 % (moderate variations), and (+++) >50 % (severe alterations) (Khalaf et al., 2019).

A scab is a hardened protective crust initiated by coagulating blood and dried tissues that develops over a wound during healing as a shield and protector; this hemostasis step establishes a part of the body's intrinsic repair mechanism. Thereafter, diverse immune cells, comprising neutrophils and macrophages, instantly invade the wound site. These cells expedite the removal of germs, debris, and damaged tissue while excreting chemical substances that augment progressive stages of healing. This inflammation stage involves inflammatory cell infiltration, which is crucial in wound healing. After that, re-epithelialization, lasting two to three weeks, consists of the mobilization and proliferation of keratinocytes from the wound margins to cover the injured area, constructing a new epithelial cell layer to restore the skin's protective barrier (Ellis et al., 2018). Subsequently, granulation tissue, recognized by a red, granular form, results from newly formed connective tissue comprised of new capillaries and small blood vessels that develop on the wound surface region during the healing process. This tissue is fundamental to supplying substantial nutrition and support for optimum wound healing. Finally, the remodeling, as fibrous scar tissue consisting of collagen, replaces regular skin following an injury. Scar tissue typically reveals less elasticity and functionality than normal tissue (Gupta and Kumar, 2015).

### 2.8 Statistical analysis

The student's *t*-test, one-way and two-way analyses of variance (ANOVA), accompanied by Tukey's *post hoc* tests, were employed to ascertain statistically significant differences among the examined samples. A significance level was established at 0.05, and (* *p <* 0.05, ** *p <* 0.01, *** *p <* 0.001, and **** *p <* 0.0001) is deemed to possess statistical significance. (Data analysis performed using Prism 6 software).

## 3 Results and discussion

### 3.1 Experimental analysis of PLO formulation

The D-optimal mixture design produced 16 trials **(**Table 3**)** to evaluate the effect of different soya lecithin, oleic acid, and pluronic ratios on the evaluated responses. Minimum levels for all independent variables were equal to one, while the maximum level for pluronic and oleic acid was 3 instead of 5 for lecithin. Regarding the selected maximum pluronic level, a low level is preferred to avoid vesicle solubilization, which results in micelle formation (Bnyan et al., 2018). Moreover, oleic acid lower-level selection considers vesicle stability and optimal membrane fluidity, as a high level can destabilize PLO structure and enhance lipid bilayer fluidity, affecting drug loading (Kundu et al., 2020). Design Expert software was employed to analyze data produced from the experimental runs using analysis of variance (ANOVA). The ANOVA results reflect statistically significant outcomes, showing different models, as demonstrated in Table 4.

**Table 3:** AML-PLOs formulation according to the Mixture design.

	Changeable inputs			Evaluated responses			
F#	Soya Lecithin ratio (X_1_)	Oleic acid ratio (X_2_)	Pluronic ratio (X_3_)	Particle size (nm) Y_1_	Polydispersity index Y_2_	Zeta potential (mV) Y_3_	Entrapment efficiency (%) Y_4_
**P1**	1.76	2.24	3.00	208.1 ± 11.4	0.4753 ± 0.043	9.46 ± 0.27	51.04 ± 3.55
**P2**	3.34	1.83	1.83	314.7 ± 12.7	0.4647 ± 0.013	9.4 ± 0.46	59.72 ± 2.99
**P3**	3.30	1.00	2.70	297.9 ± 14.9	0.4512 ± 0.023	8.42 ± 0.22	56.16 ± 3.21
**P4**	3.34	1.83	1.83	294.5 ± 11.7	0.4189 ± 0.011	8.51 ± 0.33	58.16 ± 3.01
**P5**	1.76	2.24	3.00	234.3 ± 15.7	0.4499 ± 0.022	6.78 ± 0.38	41.2 ± 4.06
**P6**	2.56	2.70	1.74	300.9 ± 10.1	0.4295 ± 0.021	8.846 ± 0.34	59.32 ± 3.27
**P7**	5.00	1.00	1.00	326.5 ± 14.3	0.4731 ± 0.024	16.11 ± 0.51	72.4 ± 4.32
**P8**	3.30	2.70	1.00	337.8 ± 13.9	0.5857 ± 0.029	7.301 ± 0.47	62.44 ± 2.12
**P9**	3.30	2.70	1.00	390.33 ± 14.5	0.556 ± 0.0278	8.777 ± 0.24	67.09 ± 2.95
**P10**	1.00	3.00	3.00	107.25 ± 5.4	0.3617 ± 0.018	5.182 ± 0.36	40.4 ± 4.02
**P11**	3.30	1.00	2.70	233.5 ± 12.7	0.4561 ± 0.021	11.74 ± 0.49	54.24 ± 3.71
**P12**	4.18	1.00	1.82	340.2 ± 14.01	0.4421 ± 0.032	17.343 ± 0.47	65.8 ± 2.29
**P13**	1.73	3.00	2.27	162.8 ± 10.14	0.36219 ± 0.02	9.52 ± 0.38	45.12 ± 4.26
**P14**	4.17	1.83	1.00	386.1 ± 14.31	0.4856 ± 0.014	16.03 ± 0.60	73.32 ± 4.67
**P15**	3.34	1.83	1.83	352.7 ± 15.6	0.4925 ± 0.024	14.24 ± 0.31	65.6 ± 1.28
P16	2.61	1.81	2.58	280.1±11.9	0.417±0.011	7.19±0.15	40.64±3.58

**Table 4:** ANOVA results for AML-PLOs formulae mixture design responses.

Response	R^2^	Adjusted R^2^	Predicted R^2^	Adequate precision	Model	F-value	*P*-value	Significance
PS (nm)	0.9124	0.8687	0.7610	16.3631	Quadratic	20.84	<0.0001	Significant
PDI	0.8252	0.7378	0.6193	10.3503	Quadratic	9.44	0.0015	Significant
ZP (mV)	0.5883	0.5249	0.3853	9.7663	Linear	9.29	0.0031	Significant
EE (%)	0.8562	0.8340	0.8022	18.4070	Linear	38.69	<0.0001	Significant

Abbreviations: PS, particle size; PDI, polydispersity index; ZP, zeta potential; EE, entrapment efficiency.

#### 3.1.1 Influence of the variable ratios on responses

##### 3.1.1.1 Particle size

AML-PLOs particle size ranged from 107.25 ± 5.4 nm to 390.33 ± 14.5 nm, as shown in Table 3**,** reflecting the nanometric range for all formulae. The model fits a quadratic model with a nonsignificant lack of fit (*p* *=* 0.7919). The following equation represents the model for PS (Y_1_):

(7) $$\mathrm{PS}=+337.83\times {\;}_{1}–102.63\times {\;}_{2}+209.11\times {\;}_{3}+862.02\;X_{1}X_{2}–35.98\;X_{1}X_{3}+198.90\;X_{2}X_{3}$$

**Fig. 1:**
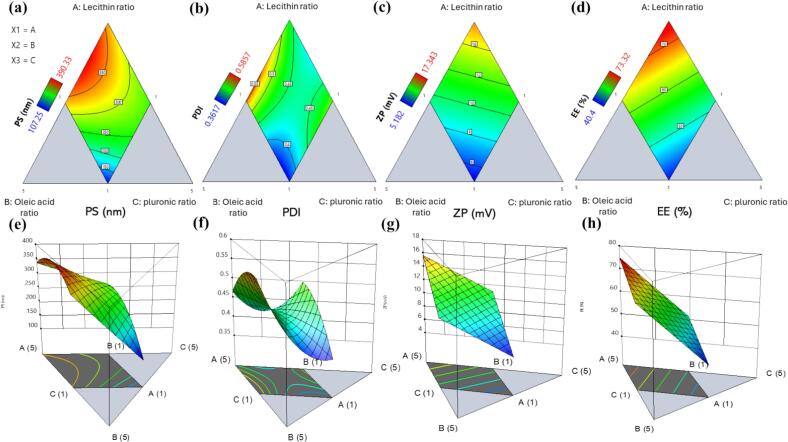
**Contour plot** of the influence of variables on (a) Particle size (PS), (b) Polydispersity index (PDI), (c) Zeta potential (ZP), and (d) Entrapment efficiency (EE), **3D plots** upon the investigation of (e) Particle size (PS), (f) Polydispersity index (PDI), (g) Zeta potential (ZP), and (h) Entrapment efficiency (EE); results represent the significant impact of different variables ratios on all responses.

As illustrated in Fig. 1a,e, an increase in lecithin level (X_1_) positively affected particle size. Soya lecithin is composed of several phospholipid molecules, and upon increasing concentration, a larger bilayer structure is formed (Faller, 2020). Otherwise, oleic acid (X_2_) negatively affected vesicle size, as can be interpreted by the capability of monounsaturated fatty acids (OA) to integrate into the lipid bilayer and regulate its rigidity and fluidity (Dragicevic et al., 2015). Moreover, oleic acid induces curvature of a lipid layer upon altering membrane shape and bending, and it can disrupt large vesicles to promote the formation of small, stable vesicles (Kundu et al., 2020).

Furthermore, A positive relation with PS was revealed regarding the effect of pluronic ratio (X3); the micelle theory is answerable to pluronic attributes, as a high SAA ratio is able to generate micelles that can be incorporated into the vesicle structure, leading to size enhancement (Naharros-Molinero et al., 2022).

The interaction between soya lecithin and oleic acid ratio (X_1_X_2_) significantly and positively affected PS (*p* *=* 0.0095). The synergistic effect of the lecithin-oleic combination can establish a balance between vesicle structure stability and fluidity, increasing vesicle size. Pluronic also amplifies this effect by stabilizing the system (Okuro et al., 2018). The preferred vesicle size in topical drug delivery systems is between 50 and 500 nm, with a preference for smaller sizes up to a specific limit for targeting deep dermal layers. PS minimization is desired to improve topical penetration across skin layers and ensure system efficacy and stability (Okuro et al., 2018).

##### 3.1.1.2 Polydispersity index

AML-PLOs polydispersity index values ranged from 0.3617 ± 0.018 to 0.5857 ± 0.029, as represented in Table 3. The model fits a quadratic model with a nonsignificant lack of fit (*p* *=* 0.3252), and the model's representative equation for PDI (Y_2_) is displayed as follows:

(8) $$\mathrm{PDI}=+0.4669\times {\;}_{1}+0.3951\times {\;}_{2}+1.03\times {\;}_{3}+0.4891\;X_{1}X_{2}–0.9893\;X_{1}X_{3}–1.38\;X_{2}X_{3}$$

Uniform distribution of vesicle size, represented by low PDI, upholds system stability and vesicle formulation reproducibility. Additionally, size uniformity maintains drug loading and release for attaining predictable and safe therapeutic outcomes (Danaei et al., 2018). Fig. 1**.b,f,** Clarify the effect of different OA, SL, and Pluronic ratios on PDI. Lecithin ratio (X_1_), Oleic acid ratio (X_2_), and Pluronic ratio (X_3_) each had a positive effect on PDI (*p* *=* 0.0034). Lecithin possesses a complex composition of several phospholipids, primarily phosphatidylcholine, characterized by differing hydrocarbon chain lengths and saturation levels. This diversity may form a less homogeneous bilayer structure and assorted-sized vesicles, increasing the PDI (Mkam Tsengam et al., 2022). As for oleic acid, the arrangement of OA within lipid bilayers can disrupt the vesicle membrane, resulting in a wide range of vesicle sizes. In addition, the impact of OA on vesicle fluidity and dynamic behavior can produce less uniform vesicles and elevated PDI values (Kundu et al., 2020).

The pluronic positive effect as an independent variable (X_3_) on PDI can be elucidated by excessive micelle formation in the aqueous solution of the vesicle structure at a high level, leading to less size uniformity. At diminished concentrations of SAA, the polydispersity index (PDI) is often lower due to the formation of more homogenous micelles. As the concentration of SAA rises, the PDI may increase due to excessive micelle formation, as it can aggregate, forming a larger structure (Singh et al., 2023). Otherwise, pluronic interaction with lecithin and OA, as represented in **Eq.**8**,** shows a significantly negative relation with PDI with *p* *=* 0.0060 and *p* *=* 0.0174 for X_1_X_3_ and X_2_X_3,_ respectively. This result concludes that a combination with a lower optimal ratio of lecithin, OA, and pluronic contributes all positive features in a lower ratio to override the heterogeneity outcomes in vesicle system uniformity.

##### 3.1.1.3 Zeta potential

AML-PLOs zeta potential fits a linear model with a nonsignificant lack of fit (*p* *=* 0.4490), and ZP exhibits values ranging from 5.182 ± 0.36 to 17.343 ± 0.47 mV, as demonstrated in Table 3. The equation symbolizes the ZP (Y_3_) model, which is shown as follows:

(9) $$\mathrm{ZP}=+15.67\times {\;}_{1}+3.31\times {\;}_{2}+6.51\times {\;}_{3}$$

ZP reflects vesicle surface charge; regardless of the sign, higher absolute values reflect higher repulsive forces between particles, upholding system stabilization by minimizing aggregation (Midekessa et al., 2020). Vesicle systems containing lecithin and oleic acid are predicted to exhibit a negative charge due to phosphatidylcholine's presence in lecithin and the carboxyl group in oleic acid. Otherwise, the positive charge on the prepared pluroleosomes may be relatively interpreted by pluronic adsorption onto the lecithin surface, as the existence of certain pluronic types (F-127) in a specific concentration can impart a positive charge to the vesicle due to pluronic interaction and surface modification (Faller, 2020). This positive charge can be intimately related to the zwitterionic nature of lecithin, which consists of a negatively charged phosphate group and a positively charged choline moiety (Parekh et al., 2023). Accordingly, the Pluronic acts independently to modify the lecithin surface charge; however, the positive charge with the higher lecithin ratio can explain the augmentation in positive ZP in a linear relation, as illustrated in Fig. 1c,g.

##### 3.1.1.4 Entrapment efficiency

AML-PLOs entrapment efficiency fit a linear model with a nonsignificant lack of fit (*p* *=* 0.4735), and measured EE% ranged from 40.4 ± 4.02 to 73.32 ± 4.67, as elucidated in Table 3. The representative equation for the EE% (Y_4_) model is defined as mentioned:

(10) $$\mathrm{EE}=+74.85\times {\;}_{1}+52.96\times {\;}_{2}+26.03\times {\;}_{3}$$

Optimum drug loading in the lipidic vesicular system controls release patterns, reinforces drug stability, and enhances drug penetration, facilitating the overall efficacy of drug delivery (Witika et al., 2021). Fig. 1d,h shows that all system compositions affect entrapment efficiency positively, independently, without interaction. Lecithin is a crucial phospholipid component that enhances the structure formation and stability of the vesicle system (Subramaniam et al., 2020). Augmentation at the lecithin level can improve bilayer formation, stimulating the vesicle's capacity to encapsulate hydrophilic and lipophilic drugs (Opatha et al., 2020).

Furthermore, OA can be incorporated into the lipid bilayer, enhancing its stability by disturbing the intensive configuration of saturated lipids. This structure adjustment inhibits vesicle aggregation and fusion, minimizing the prospect of drug leakage and preserving system stability(Shimokawa and Takagi, 2024). Additionally, OA improves vesicle fluidity and upholds the structure's integrity, rendering it less prone to rupture and preserving drug loading (Gong et al., 2024). Moreover, pluronic molecules can stabilize vesicle structure and promote drug entrapment by incorporating into the vesicle membrane, decreasing surface tension, and inhibiting vesicle aggregation (Naharros-Molinero et al., 2022).

#### 3.1.2 AML-PLOs optimization using adopted mixture design

**Fig. 2:**
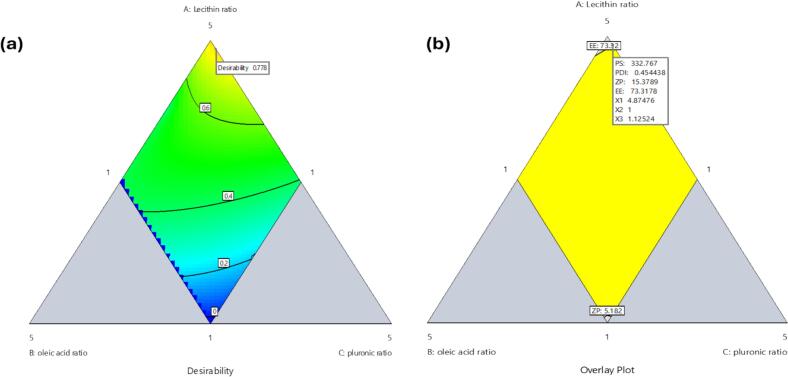
**Optimization of Mixture Design** concerning changeable inputs and desired responses; (a) desirability contour plot, and (b) graphical optimization contour plot.

The maximum desirability function for the optimum AML-PLOs system proposed by Design Expert® software was 0.778, as demonstrated in Fig. 2a**.** Optimization goals relied on minimizing PS and PDI, maximizing the absolute value of Z, and maximizing the EE percentage. The optimized selected mixture ratios comprised 4.875 for lecithin, one for oleic acid, and 1.125 for pluronic. Consequently, it was formulated and evaluated. The observed responses of the experimental trial were 320.56 ± 15.5 nm, 0.4461 ± 0.03, 15.261 ± 0.62 mV, and 71.25 ± 3.52 %, showing good correlation with predicted values (Fig. 2b**)** as PS, PDI, ZP, and EE were 332.767 nm, 0.454, 15.379 mV, and 73.32 %, respectively. The selected optimized AML-PLOs formulation showed a drug loading of 64.5 ± 0.874 %.

### 3.2 Features of optimum AML-PLOs

Subsequent analysis was applied to the optimized AML-PLOs formula, which obtained the highest desirability and fulfilled targeted features.

#### 3.2.1 Vesicle morphology

**Fig. 3:**
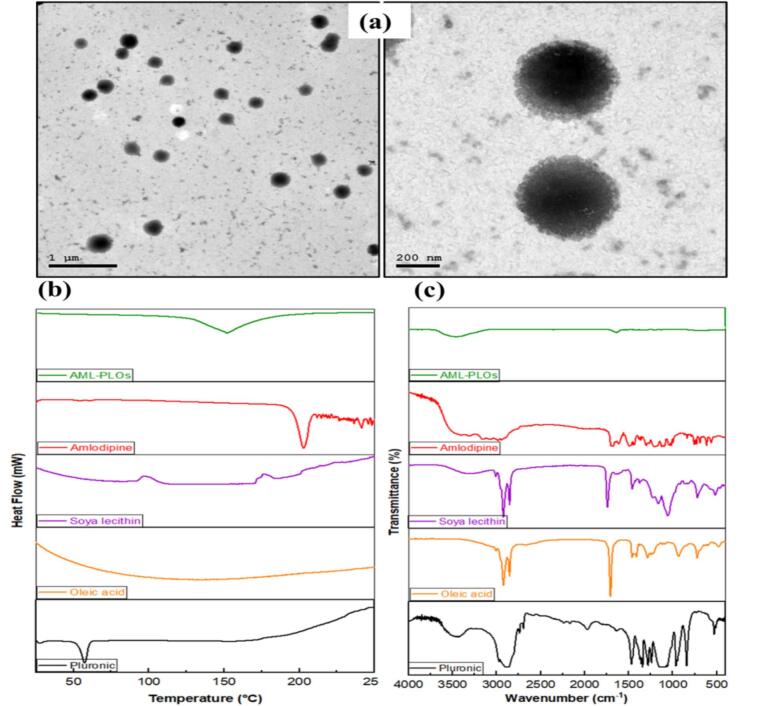
**Further assessment of the optimal AML-PLOs:** (a) TEM image of AML-PLOs, revealing uniform spherical vesicles in the nanometric range, (b) Differential scanning calorimetry thermograms, and (c) Fourier transform infrared spectra for free amlodipine, soya lecithin, oleic acid, pluronic, and optimum AML-PLOs formula; provide evidence of effective drug loading and vesicle formation.

The TEM image analysis of the optimal AML-PLO, as shown in Fig. 3a, revealed a distinct spherical vesicle with a regular size distribution. The image demonstrated that the optimum formulation size was within the nanometric range, consistent with the Zetasizer's readings.

#### 3.2.2 Differential Scanning Calorimetry

DSC thermograms were performed to ascertain AML-excipients' physicochemical compatibility and track any alterations. Fig. 3b displays that the DSC thermogram revealed an endothermic peak at about 210 °C for pure AML, which reflects its crystalline nature and matches its melting point (Hadžidedić et al., 2014). On the other hand, the AML peak's departure from the AML-PLOs thermogram proved the drug solubilization in the dispersion system and its transition from the crystalline to amorphous form (Alhamdany and Abbas, 2018).

#### 3.2.3 Fourier transform infrared spectroscopy

FT-IR established a viable approach for confirming drug solubilization and loading in the vesicle system by tracking AML characteristic peaks. FT-IR spectra of Amlodipine, Soya lecithin, Oleic acid, Pluronic, and optimized AML-PLOs formulation were shown in Fig. 3c. Pure AML's FT-IR spectrum showed many peaks, primarily due to OH (hydroxyl group) at around 3037 cm^−1^, and a band at 3352 cm^−1^ that was ascribed to N—H groups. The stretching of the C=O is associated with the band measured at about 1635 cm^−1^(Ahmed et al., 2022). The drug-effective solubilization within the PLO system was proved by the disappearance of the drug bands from the AML-PLOs spectrum. Moreover, fading other excipients' distinctive peaks in the AML-PLOs spectrum confirmed the pluroleosome fabrication with efficient loading.

### 3.3 Foamable AML-PLO system formulation

#### 3.3.1 AML-PLOs-Foam system fabrication

Different nonionic surfactants were tested to pick the formulation that acquired higher foam stability features. Tween 20, Tween 80, Brij 56, and Brij 96 were selected considering elevated HLB values to assist foaming formation capability synergistically with the anionic surfactant (SDS). All other components and conditions were constant, which indicated that any significant change in foam assessment results only refers to the impact of the anionic SAA type.

#### 3.3.2 AML-PLOs-Foam system assessment

##### 3.3.2.1 Foam calculated parameters and half-life

**Fig. 4:**
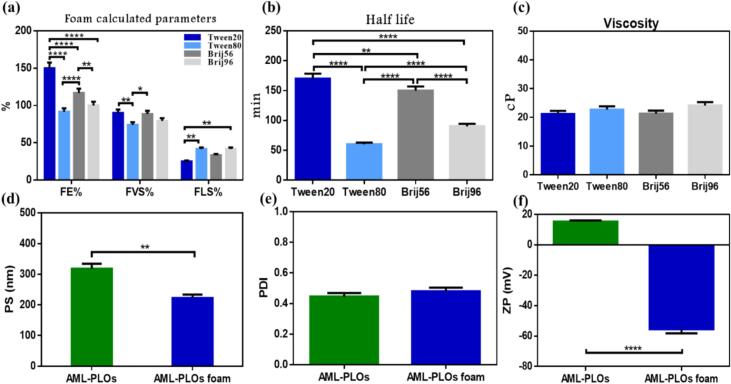
**Foam assessment** considering different types of nonionic SAA: (a) Foam calculated parameters, (b) Half-life, (c) Viscosity, **Comparative analysis** of AML-PLOs *versus* AML-PLOs foam system measuring (d) Particle size, (e) Polydispersity index, and (f) Zeta potential; revealed significant changes in PS and ZP with foam system preference.

Tween 20 possessed the highest percentage of FE and FVL, with the lowest FLS, as displayed in Fig. 4a**;** the variation is significant for all parameters compared to Tween 80 and Brij 96. FE% expresses the system's ability to expand, which indicates a more foamable formula. Otherwise, FVS and FLS are related to overall stability as higher FVS defines maximum foam stability, while FLS enhancement refers to drain stability, which opposes system stability (Falusi et al., 2024). The preference of Tween 20 in foaming capacity and foam stability can be interpreted by SAA hydrophilic-lipophilic balance (HLB) as an augmentation in HLB value results in a diminution in interfacial tension between gas and liquid (Wang et al., 2017b). The HLB values of SAA used were 16.7, 15, 12.9, and 12.4 for Tween 20, Tween 80, Brij 56, and Brij 96, respectively.

However, both types of Brij reflected preferable results than Tween 80, which can be explained by molecular structure and interfacial properties. Brijs consists of longer hydrophobic tails that produce a more elastic and robust film at the air-water interface, enhancing stability (Chouhan et al., 2024). Brij 56 outcomes (Fig. 4a) showed an insignificant difference from Tween 20, except for FE, which can be explained by considering both theories of HLB and molecular structure.

Foam half-life is fundamental for assessing the stability and efficacy of foam-based drug delivery systems (Han et al., 2020). Half-life results depicted in Fig. 4b uphold the inference of the studied calculated parameters, as Tween 20 with the higher HLB value achieves a significantly higher half-life time.

Therefore, Tween 20, with the higher foamability and stability attributes, was selected as the nonionic SAA in the foam formulation for further studies alongside the other specified compositions (SDS, HPMC, propylene glycol, and glycerine).

##### 3.3.2.2 Foam viscosity

As presented in Fig. 4c, viscosity appears with no significant modification upon the change in the nonionic SAA. HPMC is the main factor responsible for foam viscosity; nevertheless, the surfactant concentration, instead of the SAA type, can dominate the overall foam viscosity (Eita et al., 2022b). Generally, viscosity is a crucial feature in the foam formulation as it manipulates foamability and stability. For this reason, an optimal average viscosity value is pivotal, as a higher value upholds foam stability but up to a specific limit since, at the same time, further increments can hinder foam generation(Bureiko et al., 2015).

### 3.4 Optimum AML-PLOs foam evaluation

#### 3.4.1 AML-PLOs and foam system comparative analysis

Specific characteristics were evaluated to verify the suitability of the AML-PLOs foam system and to ensure the system's applicability as a convenient topical dosage form. The lipid vesicle particle size significantly affects drug transport to the skin layers. Nanosystems with a z-average around 300 nm or below can deliver the encapsulated system into deep dermal layers, while diameters above 600 nm are powerless (Danaei et al., 2018). As illustrated in Fig. 4d**,** both AML-PLOs and AML-PLO foam systems PS are suitable for application, with a significant preference for foam systems due to particle size reduction. The polydispersity index for both studied formulae, shown in Fig. 4e, represents an insignificant change with acceptable uniformity and size distribution, as it did not exceed 0.5 (Hoseini et al., 2023). Regarding zeta potential, the system surface charge is significantly inverted from positive in AML-PLOs to a highly negative charge in the foam system **(**Fig. 4f**)**, which can be interpreted relatively by the presence of SDS as an ionic surfactant in the foam composition (Quinn et al., 2020). The resulting negative charge is favorable for transdermal drug delivery as repulsive forces with the skin charge exaggerate drug penetration into skin layers (Kang et al., 2024).

#### 3.4.2 Prediction of foam bubble size

**Fig. 5:**
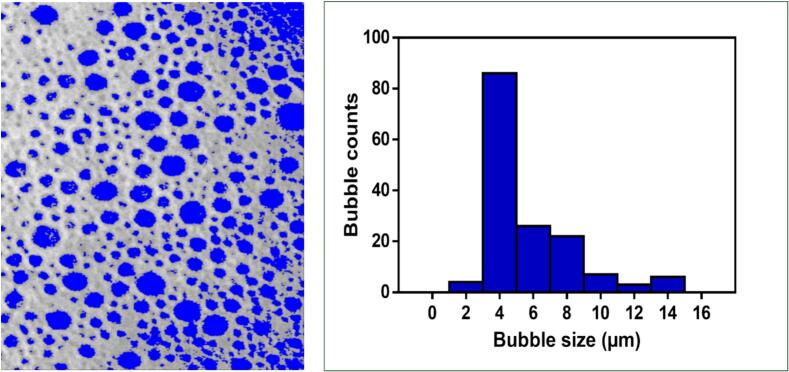
ImageJ software facilitates the estimation of bubble size and the distribution observation.

As displayed in Fig. 5, upon analyzing the AML-PLOs foam bubble image employing ImageJ software, the histogram revealed tiny, monodispersed bubbles with a majority of 4–8 μm in size. Bubble size and distribution influence foam application due to their impact on rheological and stability patterns. As noticed by Mirtič et al., the bubble distribution effect is forceful as the disparate arrangement destabilizes the foam. Otherwise, the elevated concentration of SDS may lead to diminishing bubble size with elevated homogeneity (Mirtič et al., 2017).

#### 3.4.3 Storage impact on PS, PDI, and ZP

**Fig. 6:**
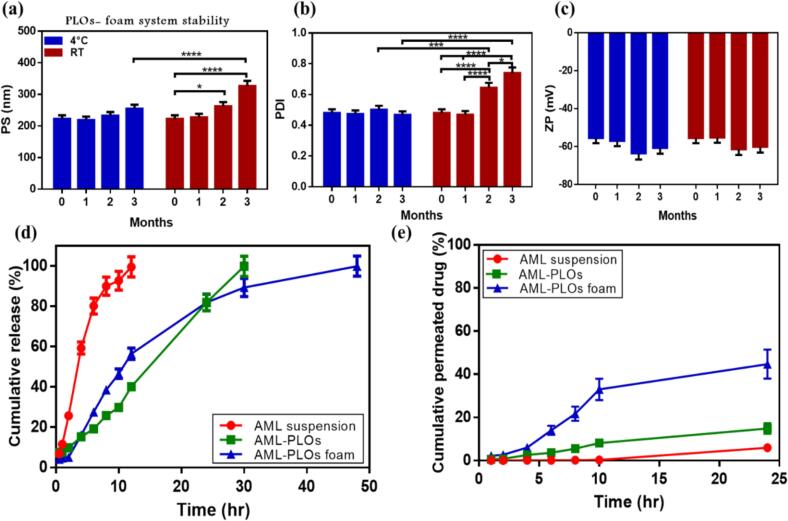
**Stability upon storage for 3 months**: studied sample storage room temperature (RT) and 4 °C & depicted by (a) Particle size, (b) polydispersity index, and (c) Zeta potential; exhibits stable properties with no noticeable significant difference comparable to fresh sample when stored at 4 °C. (d) ***In-vitro* release study** and (e) ***Ex-vivo* permeation study** illustrated the cumulative proportion of amlodipine drug suspension compared to optimized AML-PLOs and AML-PLOs foam system.

Upon storage, the PLO foam system was revealed to conserve its milky appearance without remarkable phase separation or aggregation, as manifested by visual inspection for 3 months. However, the formula performs properly upon foam fabrication by utilizing the actuator. Compared to the fresh sample, assessed values showed insignificant changes (*p* > 0.05) upon storage at 4 °C, as presented in Fig. 6a-c. Stability aspects may be upheld by the foam system components, which comprise different surfactants and stabilizing agents. A surfactant mixture in a foam system establishes a stable formulation by adsorbing and reducing interactions at the interface between different phases to avoid separation and aggregation (Kaur et al., 2024). Furthermore, the system's higher negative charge upholds the electrostatic repulsion stabilization effect (Ly et al., 2024). Otherwise, storage conducted at room temperature significantly affects the PS and PDI of the foam system, as shown in Fig. 6a,b. Elevated temperature augments kinetic energy, hence accelerating movement and facilitating faster coalescence. Moreover, the elevated temperature reduces system viscosity, which also accelerates the coalescence rate and decreases system stability (Wang et al., 2022). Such outcomes verify the stability of the AML-PLOs foam system preferred storage condition at 4 °C, which showed no noticeable changes.

#### 3.4.4 *In-vitro* release study

The release pattern of AML suspension, optimized AML-PLOs, and AML-PLOs foam system is illustrated in Fig. 6d. Suspended AML exhibited a rapid release pattern, with about 99 % released within 12 h. Conversely, AML-PLO vesicles demonstrated a slower release, with only 40 % of the drug released after 12 h, followed by completion achieved at about 30 h. The AML-PLO formula exhibits a sustained release manner compared with the AML suspension. PLO structure forces the lipophilic drug entrapped in the phospholipid bilayer to diffuse from the lipid core before attempting to release; this result aligns with that published by Abdelalim et al. while studying drug release from the oleosome system (Abdelalim et al., 2020). Regarding the foam system, a complete release is reached after roughly 48 h in a highly controlled manner. The foam system's capacity to establish a barrier that impedes the drug release may elucidate the release pattern. Moreover, the surfactants and stabilizers employed in foam formulations may interact with vesicle membranes, modifying their permeability (Kumar et al., 2022).

The release performance of AML suspension is pertinent to the Hixson-Crowell kinetics model, which was selected as the optimal fitting model based on a higher R^2^ value of 0.9874. This model is appropriate for systems where drug particles gradually dissolve, reducing size and surface area (Talevi and Ruiz, 2022). AML-PLOs released in this study follow the Korsmeyer-Peppas model with an R^2^ of 0.9855. This model is convenient for systems when the release encompasses both diffusion and erosion, which may be observed due to the prepared vesicle components (Talevi and Ruiz, 2021). Otherwise, the Hixson-Crowell was the best-fit model for the AML-PLOs foam system with a higher R^2^ of 0.9973, which means that incorporating AML-PLOs in the foam system affected the drug release mechanism and kinetics. In the foam system, AML release is mainly controlled by vesicle dissolution, and Hixson-Crowell is inventive in representing dissolution-controlled release kinetics(Contri et al., 2021).

### 3.5 *Ex-vivo* studies

#### 3.5.1 *Ex-vivo* permeation and deposition study

AML suspension, optimized AML-PLOs, and the AML-PLOs foam system were assessed using an *ex-vivo* skin permeation study to ensure foam preference in targeting deep skin infection. As shown in Fig. 6e, the cumulative percentage of AML permeated from the drug suspension after 24 h did not exceed 6 %. Otherwise, the cumulative amount of AML permeated from PLOs and PLO foam systems at the end of the 24 h was about 15 % and 45 %, respectively. The permeation profile exhibited a distinct lag period for AML-PLOs and AML-PLOs-foam, estimated to be roughly about 3 h. The permeation flux (Jss) was 0.00499, 0.0124, and 0.0373 mg/cm^2^.h for AML suspension, AML-PLO, and AML-PLOs foam system.

Vesicle systems can augment transdermal drug absorption by mimicking the stratum corneum (SC) lipid matrix due to its lipidic composition; also, the vesicular nanometric size range can enhance drug penetration (Adel et al., 2023). Additionally, oleic acid in pluroleosome formation assists as a permeation enhancer by disrupting SC lipid structure(Raj et al., 2021). After the loaded PLOs were incorporated into the foam system, a noticeable enhancement in AML permeation was observed due to the synergistic effect of the unique features of vesicles and the foam system. Foam system composition established a critical role in promoting penetration, whereas propylene glycol acts as a permeation enhancer and glycerine as a moisturizing agent, adjusting permeation by dermal hydration (Björklund et al., 2013; Dragicevic et al., 2015). Moreover, the foam's ability to disseminate readily on the skin and cover a larger area than other formulations facilitates prolonged contact with the skin surface. It allows uniform distribution and desirable absorption of the drug-loaded formulation (Myrzagulova et al., 2025).

Upon estimating drug deposition in the skin strata after conducting the permeation study for up to 24 h, about 6.5 % was measured for AML-PLOs compared to 11 % for the AML-PLOs foam system; these outcomes agree with the penetration pattern and uphold the controlled release system theory. The foam system contained HPMC, recognized as a thickening and bioadhesive agent that facilitates prolonged adherence of the formulation to the skin and reinforces skin deposition(Pan et al., 2023).

#### 3.5.2 *Ex-vivo* visualization study using confocal

**Fig. 7:**
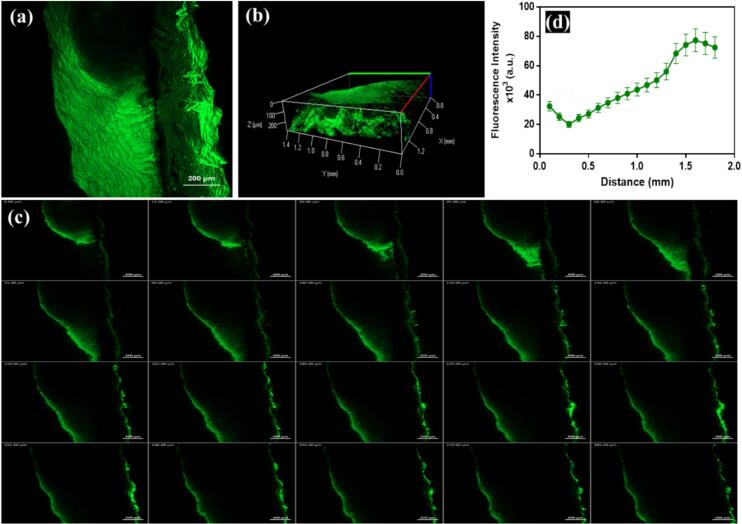
**Confocal study** of PLOs-foam system represented by (a) Tile x-y image of skin loaded with an FDA-PLO foam, (b) Three-dimensional map utilizing z-stack image of the dermal segment from 0 μm to 285 μm with an intermission of 15 μm, (c) Consecutive images from 0 to 285 μm demonstrated permeation throughout several dermal strata, and (d) Quantified assessment of FDA intensity distribution across dermal strata.

Localization of the Pluroleosome-foam system within skin layers was identified by applying a fluorescein diacetate(FDA) loaded formulation and monitoring fluorescence intensity. CLSM images represented adequate system permeation, reflected by the high dye intensity depicted in Fig. 7a-c. A quantitative assessment of accumulated fluorescence in deep dermal layers was tracked to demonstrate the depth of AML-PLO foam permeability, as shown in Fig. 7d. Various theories are proposed about nanovesicle enhancing effect on skin penetration, including the adsorption effect, trans-appendageal penetration, complete vesicle permeation due to nanometric size, and the presence of the system negative charge that influence the transcutaneous diffusion(Zong et al., 2022).

### 3.6 *In-vitro* microbiological assays

#### 3.6.1 Agar well diffusion method

**Fig. 8:**
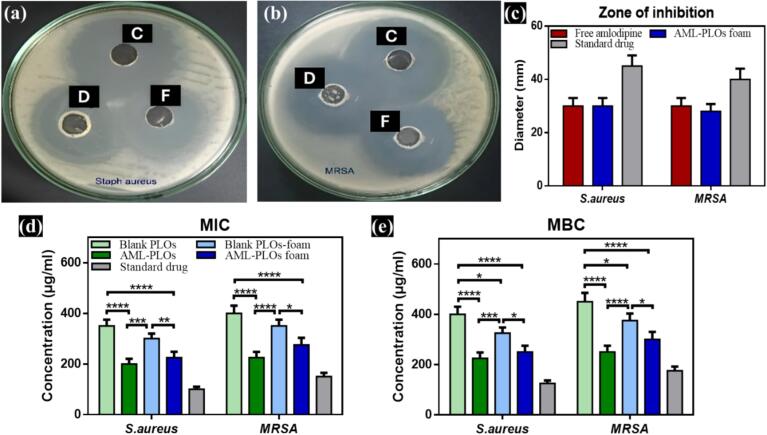
***In-vitro* microbiological assays:** Visualization of Inhibition zones on (a) *Staph aureus*, (b) MRSA, while D, F, and C symbolized amlodipine suspension, AMLO-PLOs foam system, and standard drug (Mupirax®), respectively, and results were clarified by illustrating (c) the diameter of the inhibition zone. The susceptibility of isolated bacterial species to blank PLOs, AML-PLOs, blank PLOs-foam system, AML-PLOs-foam system, and standard drug was estimated and statistically analyzed after measuring (d) MIC values, (e) MBC values.

The diameter of the growth inhibition region was depicted in Fig. 8a-c for *S. aureus* and MRSA. Regarding *S. aureus*, both free AML and AML-PLO foam systems exhibit equal sensitivity, as the measured zones for both were 30 mm. In comparison, the Mupirax® (standard drug) showed a higher value with a diameter of 45 mm **(**Fig. 8a,c**)**.In contrast, the assessment effect of the investigated formulations on the MRSA-resistant strain reflected slightly lower sensitivity to the foam-loaded formula. The growth inhibition region was 30 mm for the free AML, while the AML-PLOs-foam system showed a reduced diameter of 28 mm. Meanwhile, the standard drug recorded lower values of the inhibition zone than those of *S. aureus*, as the diameter was noticed to be 40 mm**(**Fig. 8b,c**)**.

#### 3.6.2 Micro-dilution assay (MIC/MBC estimation)

As represented in Fig. 8d,e**,** MIC and MBC values for *S. aureus* and MRSA upon exposure to blank-PLOs, AML-PLOs, blank-PLOs foam system, and AML-PLOs foam system, in addition to the standard drug (Mupirax® 2 %), were estimated and statistically evaluated for the comparison study. PLOs loaded with AML showed significantly lower MIC and MBC values than blank PLOs *(p < 0.0001);* the latter's activity against the isolated strains may be interpreted by vesicle composition, as the combination of OA and pluronic can stimulate the overall antibacterial efficacy. Kimutai et al. verified the antibacterial activity of oleic acid by discussing the inhibition mechanism of unsaturated fatty acids on *S. aureus* FabI and fatty acid synthesis (Kimutai et al., 2025). Additionally, the antiadhesive properties of Pluronic F-127 against gram-positive bacteria such as *S. aureus,* explained by Sharun et al., advocate the antibacterial efficacy as adhesion is critical to initiate biofilm development (Sharun et al., 2023). Bacteria persisting in the planktonic state are more susceptible to antibacterial agents than in biofilm states (Sharma et al., 2023)*.*

The foam system loaded with AML acquired significantly higher activity than blank-PLOs foam, as displayed by lower MIC and MBC values of loaded foam for both *S. aureus (p < 0.001) (p < 0.05)* and MRSA *(p < 0.05)*. The elevation in antibacterial activity in blank foam over blank PLOs can be explained by the existence of SDS in the foam composition. SDS can solubilize and disrupt bacterial cell membranes by denaturing proteins and enzymes, leading to cell lysis (Cho et al., 2024). Furthermore, AML-PLOs and AML-PLO foam systems showed insignificant differences between MIC and MBC values. Otherwise, as discussed before, a loaded foam system is preferred for application and penetration features.

The overall *in-vitro* microbiology study upholds the activity of amlodipine as a promising antibacterial agent; the outcomes were parallel to those noticed by Barbosa et al., which showed AML activity against *S. aureus* augmented by a synergistic effect with other antibiotics. The mechanism can be interpreted by AML disruption of bacterial cell membranes, resulting in possible cell death (Barbosa et al., 2023). In another previous *in-vitro* assessment, amlodipine inhibited several β-lactamases and was demonstrated as an antibacterial agent against resistant strains such as MRSA, especially in combination with other antibiotics (Yi, 2019).

### 3.7 *In-vivo* study

#### 3.7.1 Microbiological assays

**Fig. 9:**
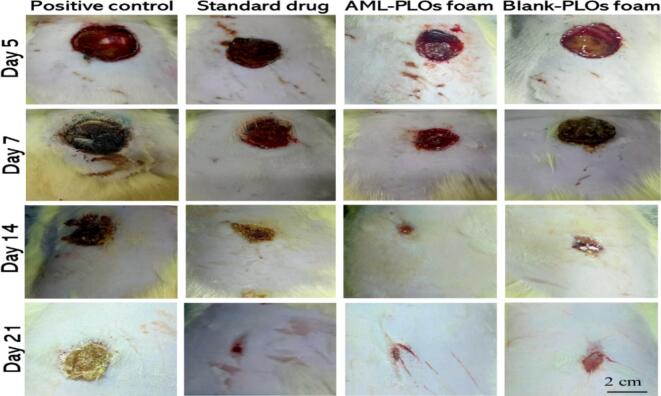
***In-vivo* open wound study:** Photographs of rat-infected wounds at 5, 7, 14, and 21 days of the four studied groups. The images demonstrate that the AML-PLOs foam formulation promotes wound healing to a degree similar to that of the standard drug (Mupirax®), suggesting its potential efficacy.

**Fig. 10:**
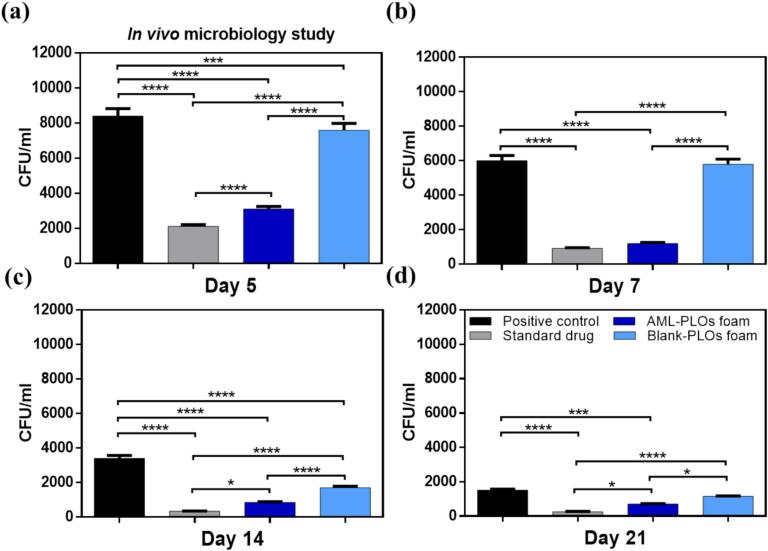
***In-vivo* microbiological assays:** MRSA counts in CFU/mL of each group observed at each time interval (a) 5 days, (b) 7 days, (c) 14 days, and (d) 21 days post-treatment. Illustrating the effect of the AML-PLOs foam system and blank-PLOs foam system compared to the standard drug (Mupirax®) and positive control.

An infected open wound rat model was adopted using MRSA strain to evaluate the activity of the prepared AML-PLOs foam and blank-PLOs foam systems in comparison to the standard drug (Mupirax®) and positive control through 21 days of treatment considering specific time intervals, as shown in Fig. 9.The observed outcomes of wound sequential images of the studied groups estimate closer manner of wound healing in AML-PLOs foam group in compare to the standard drug group and the whole study reflects preferable results of all groups over the positive control. However, microbiological counts were quantified from each group's wound tissues at different intervals to ensure activity and substantiate visual results. Figure 10a represents microbial count on day 5, as the AML-PLO foam group showed a microbial count of 3.1 × 10^3^ CFU/mL, compared to 2.1 × 10^3^ CFU/mL for the standard drug group (*p* < 0.0001), 7.6 × 10^3^ CFU/mL for the blank-PLO foam group, and 8.4 × 10^3^ CFU/mL for the positive control. On day 7, as demonstrated in Fig. 10b, The groups of blank-PLOs foam and the positive control persisted counted an elevated bacterial load, as it was 5.8 × 10^3^ and 6 × 10^3^ CFU/mL, respectively, which was significantly higher than the Mupirax®and the AML-PLOs foam groups with colony counts of 9.2 × 10^2^ and 1.2 × 10^3^ CFU/mL, correspondingly (*p <* 0.0001). The microbial count of the standard drug and AML-PLOs foam shows insignificant variation. On day 14 (Fig. 10c**)**, the AML-PLO foam group showed a reduction to 8.5 × 10^2^ CFU/mL, compared to 3.4 × 10^2^ CFU/mL in the standard drug group (*p <* 0.05). Otherwise, blank-PLOs foam was 1.7 × 10^3^ CFU/mL, and 3.4 × 10^3^ CFU/mL was observed for the positive control group. After 21 days of treatment, as manifested in Fig. 10d, the AML-PLO foam group showed an attenuation in bacterial load of 7 × 10^2^ CFU/mL, compared to 2.5 × 10^2^ CFU/mL in the standard drug group (*p <* 0.05). In comparison to the positive control, both AML-PLOs foam (*p <* 0.001) and the standard drug (*p <* 0.0001) showed a significant decrease in bacterial load. The blank-PLOs foam group was 1.2 × 10^3^ CFU/mL, which is considered a significantly higher bacterial load when compared to the results of AML-PLOs foam (*p <* 0.05) and standard drug (*p <* 0.0001).

The *in vivo* microbiology results uphold AML's antibacterial activity against MRSA, as the mechanism was explained previously in the *in vitro* study. The activity of AML as an antibacterial against *S. aureus* is assessed by previous *in vivo* studies conducted by *Andrade* et al. on the *Galleria mellonella* model, which suggests that the drug could be a promising alternative antibacterial treatment (Andrade et al., 2025). As highlighted earlier, the effect of blank-PLOs foam system composition, precisely OA, Pluronic, and SDS, appeared significantly less effective and required a more extended period. Additionally, the reduction that occurred in the bacterial load of the positive control upon completion of the study may result from the host immune response or wound environmental changes (Adib et al., 2022; Maheswary et al., 2021). However, this slight decrease did not affect wound healing.

#### 3.7.2 Wound closure

**Fig. 11:**
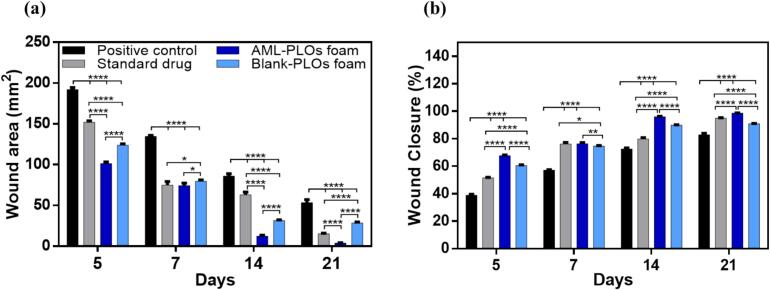
**(a) Wound areas and (b) Wound closure percentage** after different time intervals for each group after 21 days of treatment showed that AML-PLOs foam exhibited superior results compared to the standard drug, blank-PLOs foam system, and positive control.

The efficiency of the prepared AML-PLOs foam system was investigated by estimating the wound areas and the percentage of wound closure of evaluated formulae over the study-defined period, as manifested in Fig. 11**.** The AML-PLOs foam showed significantly enhanced performance of wound closure as the percentage reached about 67.7 % at the fifth day and revealed an almost 98.7 % healed wound at 21 days. In contrast, the positive control exhibited a lower rate of wound closure, about 38 % on day 5. The percentage reached 82 % throughout the experimental time, which is nevertheless considered significantly lower than AML-PLOs foam group results at all time intervals (*p <* 0.0001). Despite the lack of treatment in the positive control group, closure can be assigned to the rats' higher natural regenerative capacity. The rapid cell proliferation, accelerated tissue remodeling, and potent immune response can explain the wound closure mechanism in rats (Willyard, 2018). The standard drug group displayed amelioration in wound closure, but with a significantly lower level compared to AML-PLOs foam results (*p <* 0.0001), as it was about 51.5 % on day 5 and 95 % on day 21. In consistent with Taheri et al., the study findings represent that the lower bacterial count in treated groups results in faster wound closure (Taheri et al., 2024). Moreover, the blank-PLOs foam wound closure attributes can be interpreted by the existence of glycerin and Pluronic F-127 in the blank system. As mentioned by *Stout and McKessor,* glycerin dressing can impart a bacteriostatic environment to the wound area, leading to lower bacterial load and superior healing outcomes (Stout and McKessor, 2012). Additionally, *Sharun* et al.discuss the activity of pluronic and noticed that it can promote wound healing and form a barrier against harmful pathogens (Sharun et al., 2024).

#### 3.7.3 Histopathological dissection

Histopathological findings for the wound healing stages of the *in-vivo* study for positive control, standard drug, AML-PLOs foam, and blank-PLOs foam groups at the different studied time intervals (1,5,7,14, and 21 days of treatment) were clarified by histopathological wound healing scoring assessment as shown in Table 5. On day one, post-treatment, as demonstrated in **Fig.S1 in supplementary data**, histopathological findings for all studied groups reflect an epidermal layer disturbance at the incision margin with a significant initial inflammatory reaction comprising numerous neutrophil counts. A Fibrin clot, including red blood cells and cellular debris, was assembled, covering the wound area. Regarding the standard drug, AML-PLOs foam, and blank-PLOs foam groups, the early-stage injury and the instant inflammatory response are predominantly unaffected by topical formulations at this initial phase.

**Table 5:** Histopathological wound healing scoring assessment for positive control (G1), standard drug (G2), AML-PLOs (G3), and blank-PLOs (G4) groups for days 1,5,7,14, and 21 post-treatment.

Day	Group	Scab	Infiltration	Granulation	Scar tissue	*Re*-epithelization
**1**	**G1**	**+**	**+++**	**+**	**−**	**−**
	**G2**	**+**	**+++**	**+**	**−**	**−**
	**G3**	**+**	**+++**	**+**	**−**	**−**
	**G4**	**+**	**+++**	**+**	**−**	**−**
**5**	**G1**	**++**	**++**	**++**	**−**	**−**
	**G2**	**++**	**++**	**++**	**−**	**−**
	**G3**	**++**	**++**	**++**	**−**	**−**
	**G4**	**++**	**++**	**++**	**−**	**−**
**7**	**G1**	**++**	**++**	**++**	**−**	**−**
	**G2**	**++**	**++**	**++**	**−**	**−**
	**G3**	**++**	**++**	**++**	**−**	**+**
	**G4**	**++**	**++**	**++**	**−**	**−**
**14**	**G1**	**++**	**++**	**−**	**+**	**+**
	**G2**	**++**	**++**	**−**	**+**	**−**
	**G3**	**++**	**+**	**−**	**+**	**++**
	**G4**	**++**	**++**	**−**	**+**	**−**
**21**	**G1**	**++**	**++**	**−**	**+**	**+**
	**G2**	**++**	**++**	**−**	**+**	**+**
	**G3**	**++**	**+**	**−**	**+**	**+++**
	**G4**	**++**	**++**	**−**	**+**	**+**

The parameters mentioned above were classified as follows: (−) = normal histology (no changes), (+) ≤25 % (mild alterations), (++) = 25 %–50 % (moderate variations), and (+++) >50 % (severe alterations).

On day 5, as shown in **Fig.S2 in supplementary data,** the positive control and blank-PLOs foam groups showed persistent, significant infiltration of inflammatory cells, but there was a slight decrease compared to the first day. Improvement of granulation tissue near the wound bed, marked by new blood vessels (angiogenesis) and limited fibroblasts. Furthermore, the wound gap remains considerable, indicating the missing re-epithelialization at the wound boundary. Concerning the standard drug group, inflammatory cell infiltration was reduced, and more developed and structured granulation tissue with elevated angiogenesis and fibroblast proliferation was formed compared to the positive control. Also, a lack of significant re-epithelialization at the periphery of the wound was observed. AML-PLOs foam group revealed rapid and structured granulation tissue characterized by abundant fibroblasts and neovascularization, pointing to pro-healing activities. The tissue section of the AML-PLOs foam group indicates potentially accelerated and more extensive epithelial tissue translocation from the wound margins. The earlier positive outcomes of the AML-PLOs foam group conform with the previous assessment of Wen et al., proving that employing nanoparticles in the therapeutic strategy of infected wounds enhances therapeutic outcomes and wound healing efficiency (Wen et al., 2015).

On day 7 (**Fig.S3 in supplementary data)**, the tissue of the positive control group formed granulation tissue characterized by an elevated level of fibroblasts and collagen; despite that, it remains immature, disorganized, and less dense. Additionally, the absence of re-epithelialization persisted at the wound margins. The standard drug group displays distinctly diminished inflammatory cells, well-organized and denser granulation tissue with elevated collagen deposition, and no noticeable epithelialization. AMLP-PLOs foam group manifested a moderate presence of inflammatory cells, indicating accomplished removal of inflammation. Detection of the formation of an advanced and well-structured granulation tissue characterized by dense, mature collagen fibers was conducted. Partial re-epithelialization was perceived at the wound boundary, and angiogenesis was decreased as the wound matured. The Blank-PLOs foam results, compared to the positive control, were markedly prolonged but may be less effective in healing than the treatment groups.

**Fig.S4 in the supplementary data** shows that chronic inflammation occurred on day 14 in the positive control group, leading to markedly protracted healing or persistent infection. Primary epithelialization was recognized, and disordered collagen bundles containing residual inflammatory cells existed. Wound closure may be less effective, leading to a more extensive scar. The standard drug group revealed minimal inflammatory cells, while epithelialization was nonexistent. Mature scar tissue was developed and exhibited highly organized and dense collagen fibers. AML-PLOs foam group manifested a reduction in inflammatory cells and vascularity. In addition to advanced remodeling featuring well-formed and mature collagen bundles that closely imitate normal skin collagen. A reinforced, re-epithelialized, well-differentiated epidermis that nearly mimics natural skin was developed. Blank-PLOs foam group results were slightly analogous to the positive control, acquiring delayed or insufficient scar maturation. The scar tissue is potentially still, to some extent, undeveloped and chaotic.

**Fig. 12:**
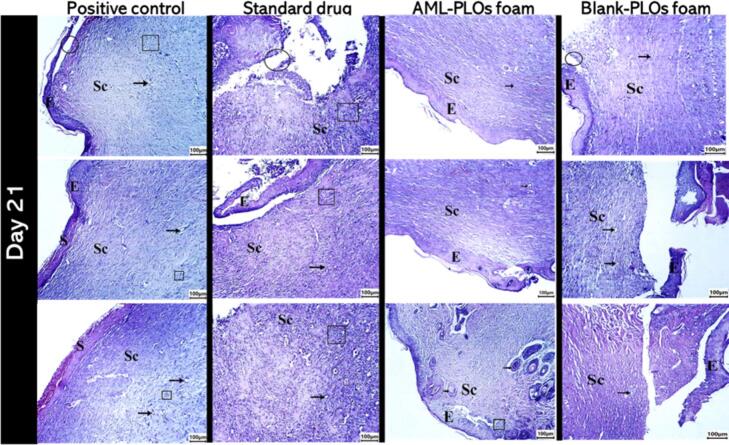
**Histopathological evaluation on day 21:** The positive control group wound was partially healed. Collagen fibers in the scar tissue (Sc) were more densely packed and demonstrated a more organized form. Fewer inflammatory cells were observed (square), and the epidermis was partially restored (E). The influence of the standard drug group is primarily noted in the lack of adverse effects from infection rather than the existence of direct pro-healing effects on tissue regeneration. A moderate inflammatory cell infiltration (square) rate and partial epithelial regeneration (E) were observed. AML-PLOs foam group showed highly organized collagen fibers, and vascularity is projected to have markedly diminished. The overall tissue architecture may have a closer resemblance to normal skin. The well-formed epithelial layer (E) and hair follicles (dotted arrow) were present in the dermal layer. Blank-PLOs foam group epidermis is partially restored (E) at the wound edge (circle).

As represented after 21 days of treatment in Fig. 12**,** the positive control group's collagen arrangement was suboptimal, with the potential existence of chronic inflammatory cells or manifestation of imperfectly regulated healing. A stable neoepidermis was present, while conceivably not completely reviving normal thickness. The standard drug group showed a partial epidermis over the scar and an assembly of dense, highly organized, oriented collagen fibers similar to normal skin. The AML-PLOs foam tissue section is perceived as an entirely regenerated epidermis, and collagen fibers exhibit a highly arranged and dense structure. Potential rejuvenation of skin, hair follicles, and sebaceous glands was noticed inside the repaired area. The demonstrated features of tissue repair, comprising re-epithelialization and assembly of collagen and keratinocytes, scar formation, represent the efficiency of formulation in defeat infection and tissue repair (Mofazzal Jahromi et al., 2018; Shende and Gupta, 2020). Otherwise, the scar in the Blank-PLOs foam group remains more protrusive and unstructured than in the treatment groups (standard drug and AML-PLOs foam), indicating the intrinsic limitations of spontaneous healing without active intervention.

Overall, the AML-PLOs foam formulation reflected significantly elevated wound healing efficacy as confirmed by expedited re-epithelialization, diminished inflammatory cell infiltration, and enhanced collagen deposition. All those stages are considered necessary for the wound healing cycle. Moreover, accumulating an organized, dense collagen matrix indicates wound reconstruction and remodeling as a reflection of the final healing step, triggering wound closure (Gupta and Kumar, 2015).

## 4 Conclusion

The current research successfully developed an innovative delivery system of amlodipine besylate (AML) and demonstrated positive outcomes that validate its efficacy as an antibacterial agent. The pluroleosomes (PLOs) established a proper and effective carrier for AML dermal administration, yielding superior results. The optimized mixture ratio consisted of 4.875 for lecithin, one for oleic acid, and 1.125 for pluronic. The optimized formulation (AML-PLOs) was loaded into a foam comprised of Tween 20 as the selected nonionic surfactant with preferred foaming capacity and stability. Tracking AML-PLOs foam features upon storage for up to 3 months at 4 °C ensured system stability and manifested insignificant changes. The loading of AML-PLOs in the foam system resulted in a more controlled release manner, as the total AML released from the PLOs-foam required 48 h compared to 30 h from PLOs. In addition, the foam system induces drug permeation and deposition *via* skin tissues, as advocated by results from the CLSM visualization study, which proved the system's ability to penetrate. The reduction observed in microbial burden in the *in-vitro* and *in-vivo* microbiology studies, in addition to improvement in histopathological outcomes, consolidated the pharmacological action of AML as an antibacterial agent. The attenuation resulted in an inflammation response and elevated wound closure rates in the group treated by AML, indicating an integrated therapeutic response between antimicrobial activity and inflammation control. However, future studies covering a broad sample size for *in-vivo* evaluation and implicating long-term safety studies are in demand. Also, studying the effect of combination therapy of AML with other renowned antibiotics can provide a synergistic framework for future initiatives aimed at targeting better therapy for infected open wounds. In spite of the promising findings regarding preclinical assessments, the applicability of translating these outcomes into clinical application faces various obstacles concerning optimizing dosing, controlling the drug therapeutic index, and ensuring systemic safety, which should also be considered in future investigations.

## CRediT authorship contribution statement

**Alaa S. Eita:** Writing – review & editing, Writing – original draft, Visualization, Methodology, Investigation, Formal analysis, Data curation. **Amna M.A. Makky:** Writing – review & editing, Writing – original draft, Methodology, Conceptualization. **Asem Anter:** Methodology, Investigation, Formal analysis, Data curation. **Islam A. Khalil:** Writing – review & editing, Writing – original draft, Visualization, Project administration, Methodology, Investigation, Formal analysis, Data curation, Conceptualization.

## Funding sources

This research received no specific grant from funding agencies in the public, commercial, or not-for-profit sectors.

## Declaration of competing interest

The authors state that they have no known competing financial interests or personal ties that could have seemed to affect the work reported in this study.

## Data Availability

The data presented in this study are available on request from the corresponding author.
